# ICT Training Perception of Professionals in Functional Diversity in Granada

**DOI:** 10.3390/ijerph20032064

**Published:** 2023-01-23

**Authors:** Carmen del Pilar Gallardo-Montes, María Jesús Caurcel-Cara, Emilio Crisol-Moya, Paula Peregrina-Nievas

**Affiliations:** 1 Department of Didactics and School Organization, University of Granada, 18071 Granada, Spain; 2 Department of Developmental and Educational Psychology, University of Granada, 18071 Granada, Spain

**Keywords:** attention to diversity, functional diversity, ICT, digital training, perceptions, professionals

## Abstract

Training in Information and Communication Technologies (ICTs) has become very valuable in the current educational panorama, given the proliferation of digital resources available in the formal and non-formal education context. Fortunately, the field of attention to diversity has also benefited from it. For this reason, it is essential that the professionals who attend to people with functional diversity have a good attitude towards them, as well as training in accordance with their requirements. The aim of this study was to analyse how professionals in Granada (Spain) perceived their ICT training. A total of 404 specialists who worked in the field of attention to diversity were interviewed. In general, participants showed a favourable opinion towards ICT but they expressed a medium-low perception of their digital training. Nevertheless, these results are encouraging, as younger teachers showed a more favourable opinion and training towards ICT. In the long term, this will be a generation that is more educated and aware of the benefits, applicability and usefulness of these resources for working with people with functional diversity.

## 1. Introduction

Teacher training in Information and Communication Technologies (ICTs) has been questioned in the last decade due to the incessant change that the educational field has undergone towards a more personalised and innovative teaching model supported by digital resources. In this way, ICTs are conformed by tools such as laptops, digital tablets, mobile phones, virtual platforms, videoconferencing platforms, applications, etc., that are used at any educational level and modality [1,2] with the aim of generating and sharing information.

Furthermore, given the considerable importance that ICT has acquired in education in the last years, teachers are also required to be trained in digital competences, defined as those “competences that 21st century teachers need to develop in order to improve their educational practice and for their continuous professional development” (p. 3) [3]. However, several studies have shown that the majority of teachers and trainee teachers have a medium-low level of knowledge of these digital options.

Numerous studies have focused on analysing the digital training and attitudes towards ICT of educational professionals in formal education [4,5,6,7,8,9,10,11] and show the most marked concerns of this group. The lack of initial training in concepts related to educational technology is highlighted [12]. In the long term, this results in teachers who are poorly trained and unaware of the didactic applications of ICT in the classroom. This shows the need to promote initial and ongoing educational training policies that include ICT training [13] and its inclusion in the classroom.

Although it is true that there is a positive perception of ICTs and their potential, teachers do not have the necessary knowledge to implement them in their daily practice [14]. Moreover, the lack of initial training is not the only concern; professional experience is also a determining factor. Those teachers with more experience in the sector often show less digital competence [15]. However, younger teachers, due to the fact that the new—and not so new—generations are “digital natives”, have a greater knowledge of the applicability and usability of these resources [15,16].

Something similar occurs with professionals working in the field of attention to diversity, not only in educational centres, but also in centres belonging to non-formal education (associations or private psycho-pedagogical therapy clinics). The training of these specialists in ICT is scarce [17,18,19,20]. Although they are receptive and motivated to broaden their knowledge of ICTs, showing favourable and positive attitudes towards them [14,21,22], they recognise the lack of training they have received [13,23].

Despite the training deficits, educational staff working in this field recognise that ICTs motivate people with functional diversity [23], promote their inclusion [23], favour the teaching-learning process, improve their cognitive development and improve their autonomy and adaptation to the environment [24].

Summarising, attention to people with functional diversity requires a transformation of the curriculum in which the attitude and training of teachers and other staff involved is essential [25]. It is important to have a properly trained professional profile with the necessary knowledge of the difficulties that technologies have for these people and thus overcome barriers to learning [20].

In a more detailed manner, Table 1 presents previous studies focused on analysing teachers’ ICT training working with students with functional diversity in different educational contexts and countries. On this point, it is worth highlighting the lack of research focused on ICT training of professionals belonging to non-formal education, finding only studies on teachers at different educational stages (Early Childhood, Primary, Secondary, Higher Education) and not on the training profile of pedagogues, psychologists, psychopedagogues, speech therapists, social educators, integration support technicians, educational monitors, etc.

For all of the reasons described above, and in view of the multitude of studies that confirm the lack of professional training in ICT in the field of attention to diversity, the aim of this research is to find out how professionals in Granada (Spain) perceive their training in ICTs, as well as their opinion about them. The objectives of this study are described below:

1. To analyse the opinion that professionals have about ICT in the field of attention to diversity.
2. To investigate the requirements and possibilities they consider ICT to have in the field of attention to diversity.
3. To determine how they perceive their training in ICT in the field of attention to diversity.
4. To determine professional performance in terms of ICT applied to people with functional diversity according to sex, gender, age, years of experience, type of institution and place of work.

## 2. Materials and Methods

The study followed a quantitative approach, with a non-experimental, contrast descriptive and transversal design [34].

### 2.1. Participants

This study involved 404 professionals from the educational and clinical fields working with people with functional diversity in the city of Granada (Spain). For their selection, non-probabilistic convenience sampling was applied [35,36].

The participants were 321 women (79.5%) and 83 men (20.5%), of whom 318 identified with the female gender (78.7%) and 86 with the male gender (21.3%). In this sense, the high number of female participants is significant, but this high representativeness was not a bias because research in the field of Social and Legal Sciences is highly feminised [37,38]. Ages ranged from 21 to 64 years (M = 39.70, *SD* = 10.95). Years of experience with people with functional diversity were grouped as: ≤5 years (48.8%), between 6 and 10 years (15.8%), 11 to 20 years (21.8%), 21 to 30 years (11.9%) and ≥31 years (1.7%). The professionals mainly worked in public schools (73.6%), subsisted schools (13.1%), private schools (2.0%), out-of-school special education centres (8.6%) or associations (7.9%) in urban (69.1%) and rural (33.6%) areas with Internet access (99.5%). Other socio-demographic data of the participants can be found in Table 2.

### 2.2. Instrument

To determine the opinion and training that the different professionals had about ICT for the care of people with functional diversity, as well as the requirements and possibilities that they presented, the DPTIC-AUT-Q questionnaire (Demands and Potentials of ICT and Apps for Assisting People with Autism), validated in Rodríguez et al. [39], was administered. This instrument had a sociodemographic data section and four subscales, the first of them aimed at the use of ICT by professionals working with people with functional diversity (which is the focus of this article) and the other three focused on the use of ICT and apps for assisting people with autism.

The subscale 1, entitled “Opinion, training and uses of ICT by professionals for teaching people with functional diversity” was a Likert scale (1 = Completely disagree; 5 = Completely agree). It had three dimensions: D1: Opinion on ICT for people with functional diversity (items 1–11); D2: Requirements and possibilities of ICT for teaching people with functional diversity (items 12–16); D3: ICT training of professionals for assisting people with functional diversity (items 17–22).

The questionnaire has adequate psychometric properties. It obtained excellent Intraclass Correlation Coefficients in Subscale 1 = 0.986 and significant Kendall’s W inter-rater concordance (*p* < 0.001) = 0.153 clarity; 0.150 coherence; 0.200 relevance; and 0.211 objectivity. The results of the CFA for Subscale 1 were equally favourable and acceptable [40,41]: the chi-square value was statistically significant (*χ^2^* = 1592.286, *p* = 0.0000). All other values indicated an adequate instrument fit: RMSEA (0.001) and WRMR (1.039), demonstrating the goodness of the model. Cronbach’s coefficient was high for each factor (α__D1_ = 0.95; α__D2_ = 0.75; α__D3_ = 0.91), as was Composite Reliability (*CR*__D1_ = 0.93; *CR*__D2_ = 0.66; *CR*__D3_ = 0.88).

### 2.3. Procedure

The study was approved by the Ethics Committee on Human Research [2002/CEIH/2021] of the University of Granada (Spain). The questionnaires were administered between May and December 2021 using the Lime-Survey platform. Contact was made by telephone, email and face-to-face at educational centres in Granada that were attended by students with functional diversity, as well as associations that support and care for these people. They were asked to collaborate voluntarily and the objectives of the research were explained to them, guaranteeing the anonymity and exclusivity of the data.

### 2.4. Data Analysis

Data were analysed with the statistical package IBM SPSS software v.28.0 for Windows, provided by the University of Granada, Granada, Spain. Descriptive statistics (mean, mode and standard deviation) and frequencies were calculated.

After checking for non-normality of the data using the Kolmogorov-Smirnov test (<0.05), non-parametric tests were applied.

First, correlation analyses were done using Spearman’s correlation coefficient [42,43], comparing and relating the three questionnaire dimensions with the participant’s age and years of professional experience. Secondly, non-parametric inferential analyses were used in order to identify whether there were significant differences between the dependent variables. To examine comparisons between the dichotomous variables “sex” and “gender”, we performed the non-parametric *U*-Mann Whitney test and estimated the effect size by calculating Cohen’s *d* [44]. For the variables “age”, “type of institution”, “place of work” and “years of experience with people with functional diversity”, we applied the Kruskal-Wallis test and the subsequent Games-Howell post hoc test, as well as the effect size using Hedges’ *H* [45].

## 3. Results

In Table 3, according to the mean and mode values, we can see that the opinion of the participants on ICT for people with functional diversity and their implementation in their professional work was located between options 4 (“Agree”) and 5 (“Completely agree”).

In Dimension 1, the averages showed by participants were high and no item reached “complete agreement”. The majority of participants agreed that the use of ICT for people with disabilities improved their own competences, provided greater flexibility in the teaching-learning process and increased the motivation of these people. However, there was less agreement that these resources were easy to use in the field of attention to diversity or that they offered multiple job opportunities for people with disabilities.

Dimension 2 followed the same trend as Dimension 1, with considerably high averages. Participants highlighted a high level of agreement that ICTs required greater investment by the different administrations, as well as specific training for it use, and they helped to provide a better attention to diversity. However, there was less agreement on knowing how to select specific ICTs according to the needs of different people with functional diversity.

In Dimension 3, the averages were lower than in the previous ones. This dimension, related to the participants’ ICT training, showed that participants did not feel competent enough to design activities with a specific software and were not prepared to help and provide support with ICT. In contrast, there was more agreement that ICT facilitated the activities’ design and adaptation and helped in the evaluation process.

The opinion on ICTs (D1) and about the requirements and possibilities (D2) that these offered to people with functional diversity obtained higher averages than Dimension 3, focused on the training that the participants had about ICT for the care of people with functional diversity (Table 4).

Correlation analysis (Table 5) showed that there was a positive, considerable (r = 0.510 to 0.750) and significant (*p* < 0.01) relationship between the opinion about ICTs and the possibilities offered by them. The same trend was observed between opinion and training (r = 0.573; *p* < 0.01). However, there was a positive relationship, average (r = 0.110 to 0.500) but significant (*p* < 0.01) between ICT opportunities and training.

No relationship was shown between the different dimensions and age or experience years with people with functional diversity.

Inferential analyses showed differences according to the sex and gender of the participants on item 5: “Are easy to use in attending to diversity” (*U* = 11,278.50; *p* = 0.020; *d* = 0.28) in favour of women (*M*_W_ = 3.88; *SD*_W_ = 0.85; *M*_M_ = 3.64; *SD*_M_ = 0.88) with a size of medium effect (*d* > 20)*;* and on items 10: “Make access to information possible” (*M*_W_ = 4.39; *SD*_W_ = 0.73; *M*_M_ = 4.57; *SD*_M_ = 0.67; *U* = 11,460.50; *p* = 0.025; *d* = 0.26) and 20: “I feel prepared to help them in the use of technical aids and use of ICT” (*U* = 11,313.00; *p* = 0.023; *d* = 0.30) in favour of men (*M*_W_ = 3.38; *SD*_W_ = 0.99; *M*_M_ = 3.66; *SD*_M_ = 0.89; *d* > 20).

The variable “age” showed statistically significant differences (Table 6). On its majority, the older group of professionals (51–64 years old), according to the Games-Howell post-hoc contrasts, showed less agreement in terms of their opinion about ICTs, the possibilities they offer and the training they have, compared to the younger group of specialists (20–30 years old) (items 7, 9, 10, 11, 12, 16, 17, 19, 20, 21 and 22), with a small effect size according to Hedges’ H-values (ε2 = 0.04). Even for items 9, 12, 20 and 21, the younger group of participants showed higher agreement than those aged 41–50, with a small effect size.

Also, “years of experience” working with people with functional diversity showed statistically significant differences. Those professionals with more experience (>31 years) were less likely to agree that ICT offered multiple opportunities in the field of attention to diversity (*M*_>31 years_ = 3.88; *SD M*_>31 years_ = 0.85), compared to those with between 21 and 30 years of experience (*M*_21 and 30 years_ = 3.88; *SD*_21 and 30 years_ = 0.85) (item 7: *K* = 10.405; *p* = 0.034), with a small effect size (ε^2^ = 0.04). Likewise, those with more experience obtained lower means than the rest for the item indicating that ICT required more material resources and investment (item 14: *K* = 10.832; *p* = 0.029), with a small effect size (ε^2^ = 0.03).

The variable “place of work” (rural, urban or both) revealed statistically significant differences in favour of rural participants in terms of ICT requiring more effort and dedication (item 12: *M*_rural_ = 4.06; *SD*_rural_ = 0.97; *M*_urban_ = 3.81; *SD*_urban_ = 0.96; *M*_both_ = 3.80; *SD*_both_ = 1.40; *K* = 6.224; *p* = 0.045), with a small effect size (ε^2^ = 0.02).

The “type of institution” (public, subsisted, private, special education centre or association) also showed differences. Specialists belonging to private institutions were less likely than the rest to agree that ICT favoured inclusion (item 6: *M*_public_ = 4.08; *SD*_public_ = 0.86; *M*_subsisted_ = 3.98; *SD*_subsisted_ = 1.05; *M*_private_ = 3.14; *SD*_private_ = 0.38; *M*_EE centers_ = 4.19; *SD*_EE centers_ = 0.80; *M*_asociation_ = 4.21; *SD*_association_ = 0.73; *K* = 10.306; *p* = 0.036), with a small effect size (ε^2^ = 0.03). Similarly, professionals at these institutions showed less agreement than those at public schools that ICTs offer multiple opportunities in the field of attention to diversity (item 7: *M*_public_ = 3.82; *SD*_public_ = 0.87; *M*_subsisted_ = 3.74; *SD*_subsisted_ = 0.92; *M*_private_ = 3.00; *SD*_private_ = 0.058; *M* _EE centers_ = 4.19; *SD* _EE centers_ = 0.63; *M*_asociation_ = 3.83; *SD*_association_ = 0.85; *K* = 12.715; *p* = 0.036), with a small effect size (ε^2^ = 0.03).

## 4. Discussion

Due to the fact that ICTs offer a world of possibilities in the field of attention to diversity [37], it is necessary to pay attention to the training that professionals who care for people with functional diversity have.

In general, participants had a favourable opinion towards ICT, with a high level of agreement that ICT improved professional competences [46], provided greater flexibility in the teaching-learning process and increased motivation for learning on the part of people with functional diversity [23,47]. On the other hand, they showed less agreement with regard to ease of use. This may be due to the difficulties that people with functional diversity encounter in using technological resources and the barriers to their use, as stated by Sabayleh and Alramamneh [48]. Consequently, professionals would find their use and implementation more complicated. In terms of ICT requirements, participants emphasised that ICTs required a large investment by governments, as well as specific training for their use, but that they did indeed help to provide better attention to diversity, favouring inclusion [23,49]. Related to the training that the different professionals had on ICT, they indicated that ICTs facilitated the design and adaptation of activities and helped in the evaluation process. But, on the contrary, they were less in agreement on their preparation to help them with the use of technical aids, showing less agreement on knowing how to select specific ICTs and on their knowledge to design activities with specific software. In general, Dimension 3, related to ICT training, was the one with the lowest mean (*M* = 3.66). This suggests that the training of professionals who care for people with functional diversity is medium-low, leaving place for improvement, as reflected in numerous previous studies [17,18,19,20,48,50].

As for the correlation analysis, significant relationships were found between training and opinion about ICTs, as well as between opinion and the possibilities they offer. This suggests that having more training in educational technology applied to people with functional diversity leads to having a more favourable opinion about it. However, no relationship was found between training and age or years of experience.

Women identified with the female gender showed greater agreement than men that ICT was easy to use in the field of attention to diversity. Male-identified men reported feeling more prepared than women to help people with functional diversity in the use of technical support and the use of ICTs, stating that ICTs make it possible to access information. These results did not necessarily agree with those obtained by Cabero-Almenara et al. [26], in which men reported less ICT training than women, or with Shater [33], in which women had more positive attitudes towards ICTs. The differences found in this study do not have large effect sizes, nor do the differences show up in most items, so there is more agreement with the study by Fernández-Batanero et al. [29], where it is indicated that sex/gender did not influence digital literacy.

One variable that did have a significant influence was age. In general, younger participants showed more agreement on all three dimensions of the questionnaire than older participants. This implied that younger professionals showed a more favourable view towards ICTs in the field of functional diversity, as well as a higher level of education, in agreement with Fernández-Batanero et al. [29], Mañanes and García-Martín [15] and Martínez-Rico [16]. The reason for this is that the younger generations, due to their social context and their link with ICT since childhood, had a greater knowledge of the technology and its possibilities. Similarly, in the current curricula in Spain [51], digital competence is present at all education levels, so that its use and knowledge in the classroom is part of everyday life. This reasoning should be taken with caution, given that having grown up with a multitude of digital resources does not imply being competent in them, but it does imply a greater knowledge of the variety of resources and their functionality.

In terms of professional experience, the more experienced participants (>31 years) were less likely to agree that ICT was a greater investment than the less experienced specialists. Similarly, this group of participants (>31 years old) expressed less agreement that ICT offered multiple possibilities in the field of diversity provision. It is possible that teachers with more professional experience did not find the functionality, applicability and opportunities in ICT that the younger group did. This may be due to the fact that these resources have not been part of their work during the course of their professional career.

Related to the workplace, participants from rural areas indicated that ICT required more effort and dedication. These results are justified by the study of Ortiz et al. [32], which shows that institutions in urban areas offer better training programmes. Thus, professionals in rural areas may have a lower opinion of ICT and a lower perception of their training than those in urban areas, precisely because of access to more specific training programmes.

The type of institution also made a difference. Professionals working in private institutions were less likely to agree that ICT favoured inclusion and that ICT offered multiple opportunities to work with people with functional diversity. These results show no relation with previous studies, since Fernández-Batanero et al. [13] found that in state-subsidised centres, the training of their professionals is lower.

In conclusion, it is worth it to highlight that not only the importance of including digital resources in formal and non-formal educational contexts, but also the importance of using them, given that ICTs are present in most of them. As it has been seen, in general, the opinion towards ICT is favourable and shows good predispositions on the part of the participants [14,21,22,33]. However, a medium-low perception towards ICT training for people with functional diversity has been expressed [15,17,18,19,20,48,50]. Even so, these results were encouraging, as younger professionals showed a more favourable opinion and training towards ICT than older ones. In the long term, this implies a generation that is more educated and knowledgeable about the benefits, applicability and usefulness of these resources in working with people with functional diversity.

As a prospective research and as a complement to this study, it would be interesting to know the perception of ICT training of current students of the main degrees that work in the fields of formal and non-formal education with people with functional diversity (teachers, pedagogues, social educators, psychopedagogues, psychologists, speech therapists...). This would address an essential component mentioned and emphasised in the introduction: the initial training of these professionals at the university stage. It would also be worthwhile to extend the sample to other cities and countries in order to carry out comparative studies between them in future research.

In terms of limitations, this study used a sample exclusively from the city of Granada, thus affecting any generalization of the results. To solve it, as mentioned above, it is proposed to expand the sample. In addition to the previously mentioned sampling limitation, there is the limitation of any survey or self-reported research [52,53], even with validated instruments. This could influence the veracity of the responses [54] or the possibility of generating new ideas or theories [55].

## 5. Conclusions

ICTs are part of the society we live in, which is known as the Knowledge Society or Information Society [56]. They contribute positively to the development of the global economy, social production and also unquestionably support the teaching-learning processes in formal and non-formal education [57]. For this reason, ICT inclusion in the educational and training context should not be seen as an isolated fact. It requires prior training on these resources, their potential and ways of incorporating them into daily professional practice. The incorporation of educational technology is currently seen as a necessity, as society itself perceives it as a useful and quality resource for education improvement.

In this sense, it is necessary to highlight that the simple fact of having digital tools in the classroom and resources that favour ICT-supported teaching does not guarantee the students’ learning [58] or the success and effectiveness of education [59]. ICT knowledge and the correct application of the possibilities offered by these technologies often require extensive training processes.

The field of attention to diversity, as has been detailed throughout this research, requires professionals who have enough knowledge—not only academic—so that in the teaching-learning process, any person has access to knowledge that makes them autonomous and allows them to develop in all those practices that society demands of them.

Consequently, if ICTs are a motivating element for people with functional diversity, a tool for communicative development and a resource to which most people have access, the formal and non-formal education sector should be more updated than other populations. This highlights the relevance of digital training for professionals working in the context of attention to diversity. The fact that these specialists do not have the appropriate knowledge and experience in the technological field leaves those people with more specific needs behind in a complex society that depends on ICT for areas as important as economy or medicine.

It is concluded that digital skills acquisition and development among educators is essential for their interaction and intervention with people with functional diversity. This research revealed a professional sector with a favourable opinion of ICT and knowledge of their possibilities, but with training needs in terms of their use in the field of attention to diversity. Advancing and deepening in this purpose, from their daily work, it is essential to promote this process and, in this way, to offer an inclusive and quality education in accordance with current social demands.

The study results showed that having digital training was linked to a more favourable opinion towards ICT. In this way, and by knowing the perception that professionals have of their training, measures can be taken to solve this situation. As mentioned by Saladino et al. [49] and Toledo and Llorente [20], having specific training is crucial, so it is necessary to attend to educators’ initial and continuous training for the implementation of ICT with people with functional diversity. Only by knowing the training needs of educators in Granada will it be possible to propose intervention plans in higher and permanent education that address these deficiencies.

The study results will be the starting point for undertaking teaching innovation projects that address training in digital resources in the field of attention to diversity for future professionals (teachers, speech therapists, pedagogues and psychologists), as well as for active educators.

## 6. Patents

The questionnaire “DPTIC-AUT-Q” (Demands and Potentials of ICT and Apps for Assisting People with Autism) is registered in the Territorial Registry of Intellectual Property of the Junta de Andalucía and the Ministry of Culture and Sport of Spain (Registration Number: 04/2021/4367; Application Number: RTA-02276-2021).

## Figures and Tables

**Table 1:** Previous studies on the use and perception of ICTs by teachers of people with functional diversity.

Work	Participants	Evaluation Instrument	Main Objective	Results
[13]	68 education professionals (Catalonia, Spain)	Semi-structured interview (e.g., “Do you consider that primary school teachers are aware and qualified to help students with specific disabilities to use technical support and ICT applications?”).	To understand the perception of key informants (directors, heads of studies and ICT coordinators from educational centres) on the degree of training and technological knowledge possessed by the teachers of Primary Education with respect to the use of ICT with students with disabilities.	The professionals interviewed perceive a low level of ICT training and knowledge applied to people with functional diversity. Subsidised educational institutions presented lower levels of ICT training.
[18]	425 Primary Education teachers (Andalusia, Spain)	Diagnosis and Teacher Training for the Incorporation of ICT in Students with Functional Diversity (DIFOTI-CyD).	To analyse the training level and technological knowledge that Primary School teachers have regarding the use of ICT with people with different types of disabilities.	Primary school teachers show low ICT capacity for people with functional diversity.
[23]	42 teachers (Portugal)	Five-dimensions survey: Personal characteristics; computer proficiency and pedagogical teaching; continuous training; ICT use in curriculum context; perceptions.	To analyse the perceptions of teachers who teach visually impaired students in middle school and secondary school in these reference schools of their knowledge, teaching and training in the area of ICT, as well as of the real ICT integration in the teaching and learning of these students.	Teachers working with people with functional diversity, and specifically with visual disability, are poorly trained in ICT. Teachers specialising in visual disability are more trained than generalist teachers.
[26]	1194 teachers (Early Childhood Education, Primary and Secondary Education and University) (Andalusia, Spain).	Teacher training students’ technological knowledge on the use of Information and Communication Technologies (ICT) for people with special educational needs (COTETICNE) [27].	To analyse teachers’ knowledge about digital resources to support learners with disabilities.	Teachers have a medium-low level of knowledge about digital resources to attend students with functional diversity. Gender has an influence: men show less digital competences. The stage of work is a determining factor: higher stages = lower level of digital competence.
[28]	104 University professors (Castilla-La Mancha, Spain).	Diagnosis and Teacher Training for the Incorporation of ICT in Students with Functional Diversity (DIFOTI-CyD) [29].	To determine the training level and technological knowledge that teachers have regarding the application of ICT as a support for students with disabilities.	University professors present a low level of technological training to integrate digital tools in the classroom with students with functional diversity. Gender has no influence on the level of digital competence. Professors under the age of 40 years have a higher level of digital competence.
[30]	920 teachers (Grece)	Mobile Learning Readiness Survey (MLRS).	To analyse teachers’ perceptions of preparation for mobile learning.	Teachers with less experience (younger) are more inclined to use ICT in the classroom. Teachers who use ICT more find more benefits in their job.
[31]	29 teachers from Palermo (Italy) and 11 teachers from Burgos (Spain)	Questionnaire for evaluating digital teachers’ competences.	To know teachers’ digital competences.	Italian teachers have a low digital competence to deal with students with functional diversity. The digital competence of Spanish teachers is medium.
[32]	2396 education professionals (Spain)	DISTIC5 Questionnaire.	To recognise teacher appreciation of ICT training to address diversity needs.	Educational professionals have a medium-low level of co-knowledge about digital resources for attending to pupils with functional diversity. There are better ICT training programmes in urban centres than in rural centres.
[33]	124 teachers (Irbid, Jordan)	Questionnaire on teachers’ attitudes towards digital learning in students with functional diversity.	Determine the perceptions of public school teachers about digital learning for disabled students.	Teachers’ attitudes towards ICT for pupils with functional diversity are positive. Women express more favourable attitudes than men.

**Table 2:** Other socio-demographic data of the participants.

Variables		*n* (%)
Type of professional	General teacher	142 (35.1)
	Specialist teacher	88 (21.7)
	Teacher of Therapeutic Pedagogy	87 (21.5)
	Speech and Hearing Teacher	58 (6.9)
	Special Education assistant	25 (6.2)
	Speech therapist	20 (4.9)
	Therapeutic Pedagogy and Integration class teacher [PTAI]	12 (3.0)
	Psychologist	12 (3.0)
	Special Needs class teacher [PTAE]	7 (1.7)
	Psychopedagogue	6 (1.5)
	Therapeutic companion	6 (1.5)
	Social worker	5 (1.2)
	Pedagogue	5 (1.2)
	Occupational therapist	5 (1.2)
	Social Integration Technical Specialist [PTIS]	1 (0.2)
Diversity worked with	Behavioural (attention deficit hyperactivity disorder, …)	280 (69.1)
	Development (Autism, Asperger, …)	247 (61.0)
	Oral Language (Dyslalia, …)	203 (50.1)
	Intellectual	202 (49.9)
	Written Language (Dyslexia, Dysgraphia, …)	216 (53.3)
	Sensorial (Visual or Hearing Disability)	107 (26.4)
	Physical/Motor	106 (26.2)
	Emotional (Anxiety, Stress, Depression, …)	78 (19.3)
	Multiple	58 (14.3)
	Mental Disorder (Bipolar Disorder, Schizophrenia, ...)	49 (12.1)
Stage of work	Child Labour	290 (71.6)
	Primary	180 (44.4)
	Secondary	104 (25.7)
	Adults	33 (8.1)
	Senior citizens	6 (1.5)

Note. Given as multiple response items. PTAI = (in Spanish) Maestro de Pedagogía Terapéutica de Apoyo a la Integración; PTAE = (in Spanish) Maestro de Pedagogía Terapéutica de Apoyo Específico; PTIS = (in Spanish) Personal Técnico de Apoyo a la Integración.

**Table 3:** Opinion, training and ICT requirements for people with functional diversity.

ITEM		*M*	*SD*	*M_o_*	%				
					1	2	3	4	5
**D1. Opinion**	1. Improve the competences of the teacher	4.40	0.75	5	1.0	1.0	6.9	8.9	48.4
	2. Require advice on the search for, selection and evaluation of ICT resources for the teaching-learning process	4.23	0.84	4	2.0	1.0	11.9	42.7	42.5
	3. Provide greater flexibility in the teaching-learning process	4.40	0.74	5	0.5	1.7	6.9	38.8	52.1
	4. Make it possible to meet educational needs	4.24	0.80	5	0.5	1.7	15.1	39.0	43.7
	5. Are easy to use in attending to diversity	3.83	0.88	4	0.7	5.2	28.6	41.5	24.0
	6. Enable inclusion	4.07	0.87	4	0.7	3.5	20.0	39.5	36.3
	7. Offer multiple opportunities in attending to diversity	3.82	0.87	4	1.2	5.4	24.2	48.1	21.0
	8. Improve performance and efficacy	4.02	0.80	4	0.7	1.5	22.2	46.2	29.4
	9. Increase motivation in learning	4.47	0.70	5	0.7	1.0	4.7	38.0	55.6
	10. Make access to information possible	4.42	0.72	5	0.5	1.0	7.7	37.3	53.6
	11. Make it possible to achieve aims in a more flexible way	4.20	0.78	4	0.5	2.0	13.6	44.4	39.5
**D2. Requirements and possibilities**	12. They require greater commitment and effort in my work	3.89	0.98	4	1.7	5.9	25.7	34.8	31.9
	13. They require specific training	4.35	0.79	5	1.2	1.7	7.4	39.8	49.9
	14. They need more material means and investment by management	4.50	0.72	5	0.5	1.2	6.4	31.6	60.2
	15. They help give more attention to diversity	4.39	0.79	5	0.7	2.0	9.4	33.8	54.1
	16. I would know how to choose specific ICTs according to their needs	3.55	0.3	4	2.0	9.6	35.1	38.0	15.3
**D3. Training in ICT**	17. I know the main limitations that can condition its use	3.68	0.92	4	2.0	6.9	30.1	42.7	18.3
	18. I know different internet sites where I can find specific resources	3.89	0.92	4	2.0	6.2	18.0	48.4	25.4
	19. I know how to design activities with non-specialist educational software	3.03	1.17	3	11.4	20.2	34.3	21.7	12.3
	20. I feel prepared to help them in the use of technical aids and use of ICT	3.44	0.98	4	3.0	14.3	30.9	39.5	12.3
	21. It makes it easier for me to design and adapt activities	3.92	0.82	4	0.7	3.7	22.2	49.6	23.7
	22. It helps me to carry out assessment	3.97	0.84	4	1.0	2.5	23.0	45.7	27.9

Note. *M* = mean; *SD* = standard deviation; *M_o_* = Mode.

**Table 4:** Three dimensions’ mean and standard deviation.

Dimension	*M*	*SD*
D1. Opinion	4.19	0.56
D2. Requirements and possibilities	4.14	0.51
D3. Training in ICT for functional diversity	3.66	0.73

Note: *M* = Mean; *SD* = Standard deviation.

**Table 5:** Spearman correlation between opinion on ICT, ICT opportunities, training, age of the professional and professional experience.

	D1	D2	D3	Age	Experience
**D1**	1				
**D2**	0.554 **	1			
**D3**	0.573 **	0.441 **	1		
**Age**	−0.111 *	0.047	−0.192 **	1	
**Professional experience**	−0.006 *	0.066	0.000	0.603 **	1

Note. ** The correlation is significant at level 0.01 (two-tailed); * the correlation is significant at level 0.05 (two-tailed).

**Table 6:** Significant differences according to the participants’ age.

Age Groups (Years Old)									Kruskal-Wallis	
I	20–30 (*n* = 103)		31–40 (*n* = 118)		41–50 (*n* = 109)		51–64 (*n* = 74)			
	*M*	*SD*	*M*	*SD*	*M*	*SD*	*M*	*SD*	*K*	*p*
**7**	4.01	0.87	3.71	0.91	3.87	0.80	3.65	0.85	11.54	0.009 **
**9**	4.66	0.57	4.53	0.62	4.34	0.85	4.30	0.68	17.75	0.000 ***
**10**	4.54	0.70	4.47	0.73	4.35	0.73	4.28	0.71	10.68	0.014 *
**11**	4.32	0.77	4.29	0.76	4.15	0.80	3.99	0.77	12.01	0.007 **
**12**	3.66	1.04	3.92	0.92	4.06	0.97	3.92	0.95	9.11	0.028 *
**16**	3.66	0.96	3.53	0.94	3.61	0.95	3.32	0.81	8.93	0.030 *
**17**	3.83	0.91	3.62	0.89	3.73	0.99	3.49	0.83	9.61	0.022 *
**19**	3.33	1.19	2.92	1.12	3.11	1.16	2.72	1.15	13.32	0.004 **
**20**	3.73	0.93	3.40	1.01	3.45	0.98	3.07	0.88	22.90	0.000 ***
**21**	4.07	0.86	3.92	0.82	3.92	0.78	3.69	0.76	11.82	0.008 **
**22**	4.19	0.76	4.05	0.84	3.82	0.93	3.74	0.83	19.83	0.000 ***

Note. *I* = number of item; *n* = number of elements that make up the sample; *M* = mean; *SD* = standard deviation; *U* = results of Mann-Whitney *U* test; *p* = probability associated with *U*; statistically significant: * *p* < 0.05; ** *p* < 0.01; *** *p* < 0.001.

## Data Availability

Not applicable.
